# Longitudinal outcomes of amyloid positive versus negative amnestic mild cognitive impairments: a three-year longitudinal study

**DOI:** 10.1038/s41598-018-23676-w

**Published:** 2018-04-03

**Authors:** Byoung Seok Ye, Hee Jin Kim, Yeo Jin Kim, Na-Yeon Jung, Jin San Lee, Juyoun Lee, Young Kyoung Jang, Jin-ju Yang, Jong-Min Lee, Jacob W. Vogel, Duk L. Na, Sang Won Seo

**Affiliations:** 1 Department of Neurology, Yonsei University College of Medicine, Seoul, 03722 Korea; 2 Department of Neurology, Samsung Medical Center, Sungkyunkwan University School of Medicine, Seoul, 06351 Korea; 3 Department of Neurology, Chuncheon Sacred Heart Hospital, Hallym University College of Medicine, Chuncheon, Korea; 4 Department of Neurology, Pusan National University Yangsan Hospital, Yangsan, South Korea; 5 Department of Neurology, Kyung Hee University Hospital, Seoul, Korea; 6 Department of Biomedical Engineering, Hanyang University, Seoul, South Korea; 7 Montreal Neurological Institute, McGill University, Montrèal, Quebec, Canada

## Abstract

We aimed to compare the longitudinal outcome of amnestic mild cognitive impairment (aMCI) patients with significant Pittsburgh Compound B uptake [PiB(+) aMCI] and those without [PiB(−) aMCI]. Cerebral β-amyloid was measured in 47 patients with aMCI using PiB-positron emission tomography (PET) (31 PiB(+) aMCI and 16 PiB(−) aMCI). Clinical (N = 47) and neuropsychological follow-up (N = 37), and follow-up with brain magnetic resonance imaging (N = 38) and PiB-PET (N = 30) were performed for three years. PiB(+) aMCI had a higher risk of progression to dementia (hazard ratio = 3.74, 95% CI = 1.21–11.58) and faster rate of cortical thinning in the bilateral precuneus and right medial and lateral temporal cortices compared to PiB(−) aMCI. Among six PiB(−) aMCI patients who had regional PiB uptake ratio >1.5 in the posterior cingulate cortex (PCC), three (50.0%) progressed to dementia, and two of them had global PiB uptake ratio >1.5 at the follow-up PiB-PET. Our findings suggest that amyloid imaging is important for predicting the prognosis of aMCI patients, and that it is necessary to pay more attention to PiB(−) aMCI with increased regional PiB uptake in the PCC.

## Introduction

Cerebral β-amyloidosis is a key requirement for development of Alzheimer’s disease (AD) according to the amyloid cascade hypothesis^1,2^. Amnestic mild cognitive impairment (aMCI) is regarded as a transitional state between normal aging and dementia, especially AD^3,4^. With development of biomarkers for β-amyloid, *in vivo* identification of fibrillar β-amyloid is possible in aMCI patients^5^. Previous studies have shown that about 40–70% of aMCI patients showed measurable fibrillar β-amyloidosis using Pittsburgh Compound B positron emission tomography (PiB-PET)^6–8^. Several heterogeneous possibilities have been proposed as underlying causes for PiB-negative [PiB(−)] aMCI patients including cognitive impairment related to depression, accelerated brain aging as indicated by abnormality in vascular markers^9,10^, and other pathologies such as cerebrovascular disease^3^, hippocampal sclerosis^11^ and argyrophilic grain disease^12^. In our previous cross-sectional study^9^, we categorized PiB(−) aMCI group into several subgroups including depressive aMCI, aMCI with increased PiB uptake in the posterior cingulate cortex, aMCI with small vessel disease (SVD) and aMCI with accelerated aging. The PCC-PiB(+) aMCI subgroup exhibited decreased cortical thickness in the medial temporal lobe and deformity in the bilateral hippocampus, which was similar with that in AD^9^.

Previous studies have also shown that β-amyloidosis in aMCI is predictive of future progression to dementia^8,13–15^. However, there are wide variations in the risks associated with β-amyloidosis in aMCI patients, and the prognosis of aMCI patients without β-amyloidosis has not been clearly demonstrated. Furthermore, most longitudinal studies have focused on amyloid (+) aMCI patients. In the current study, we followed aMCI patients included in the previous cross-sectional study^9^ up for three years and aimed to investigate the longitudinal outcome of β-amyloidosis in a prospectively recruited cohort of aMCI patients. We also subcategorized the amyloid-negative aMCI patients and investigated clinical features, neuroimaging and their prognosis. We hypothesized that (1) PiB(+) aMCI patients have greater risk of progression to dementia compared to PiB(−) aMCI patients; and (2) there could be certain features predicting progression to dementia in PiB(−) aMCI patients.

## Results

### Demographic features

Among 47 aMCI patients who were followed up for 3.6 years, 31 (66.0%) patients were PiB(+) and 16 (34.0%) patients were PiB(−) at baseline. The comparison of demographic and clinical features is presented in Table 1. Compared to PiB(−) aMCI patients, PiB(+) aMCI patients had lower scores in the Geriatric Depression Scale, a higher proportion of *APOE4* carriers, and a lower proportion of diabetes mellitus [one PiB(+) aMCI (3.2%) vs. 4 PiB(−) aMCI (25.0%) patients, P = 0.040 for chi-square test] and hypertension [10 PiB(+) aMCI (32.3%) vs. 10 PiB(−) aMCI (62.5%) patients, P = 0.047].

**Table 1:** Demographic features of PiB(−) and PiB(+) aMCI with clinical follow-up.

	PiB(−) aMCI	PiB(+) aMCI	*p* value
N	16	31	
Baseline age: years	71.6 ± 6.3	70.2 ± 8.7	0.570
Gender, female	9 (56.3)	11 (35.5)	0.172
Education, years	11.5 ± 6.1	13.6 ± 3.3	0.218
Geriatric Depression Scale	15.1 ± 7.7	9.6 ± 6.0	0.010
*APOE4* carrier	0/16 (0)	19/29 (65.5)	<0.001
Baseline PiB uptake	1.32 ± 0.13	2.16 ± 0.35	<0.001
Annual changes of global PiB retention ratio^*^	0.01 ± 0.04	0.03 ± 0.06	0.258
Follow-up duration, years	3.8 ± 1.5	3.5 ± 1.3	0.307
Progression to dementia	4 (25.0)	22 (71.0)	NA

AD = Alzheimer’s disease; *APOE4* = *apolipoprotein E4*; aMCI = amnestic mild cognitive impairment; PiB(−) aMCI = Pittsburgh compound B negative amnestic mild cognitive impairment; PiB(+) aMCI = Pittsburgh compound B positive amnestic mild cognitive impairment; NA = not applicable. Data are expressed in mean ± standard deviation or number (percentage). P values are results of independent t tests or chi-square tests as appropriate. ^*^Nine of 16 PiB(−) aMCI patients and 21 of 31 PiB(+) aMCI patients performed follow-up PiB-PET scans.

### Conversion to dementia

**Figure 1:**
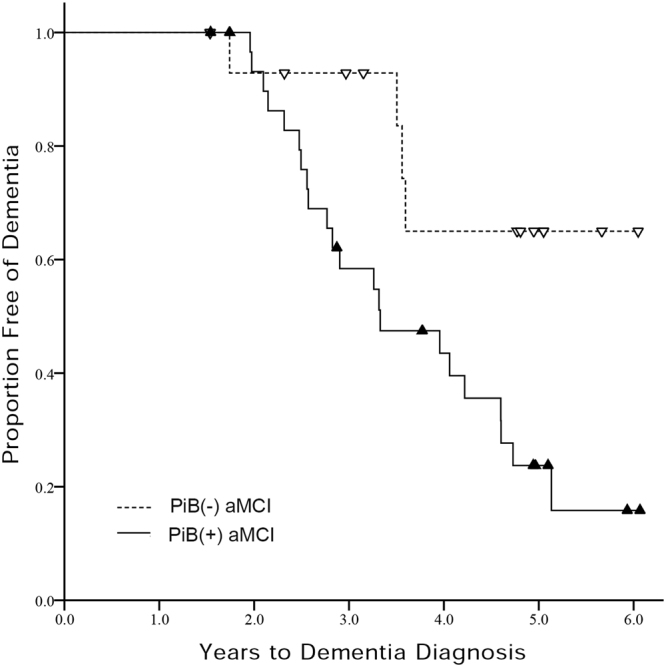
Kaplan-Meier curves for PiB(+) aMCI and PiB(−) aMCI groups. PiB(−) aMCI = Pittsburgh compound B negative amnestic mild cognitive impairment; PiB(+) aMCI = Pittsburgh compound B positive amnestic mild cognitive impairment.

Twenty-two of 31 PiB(+) aMCI subjects progressed to dementia within 3.6 years (progression rate = 20.8%/year), while four of 16 PiB(−) aMCI group progressed within the same timespan (progression rate = 6.3%/year). Figure 1 shows Kaplan-Meier survival curves for PiB(+) aMCI and PiB(−) aMCI groups. Cox regression models showed that PiB(+) aMCI had a higher risk of progression to dementia [hazard ratio (HR) = 3.74, 95% CI = 1.21–11.58] after controlling for age, sex, and education. All PiB(+) aMCI patients who progressed to dementia were diagnosed as probable AD, while PiB(−) aMCI patients progressed to various causes of dementia including probable AD (1), progressive supranuclear palsy syndrome (PSPS) (2) and subcortical vascular dementia (1).

### Neuropsychological changes

Longitudinal changes in neuropsychological test scores which were investigated using linear mixed effect models are summarized in Table 2. Linear mixed models separately performed in PiB(+) aMCI group showed that there was significant detrimental effect of time on calculation, immediate recall and delayed recall items of SVLT and RCFT, COWAT animal, stroop color reading, MMSE, and CDR Sum of Boxes scores after controlling for age, sex and education. In PiB(−) aMCI group, there was no neuropsychological test showing significant change according to the time interval from baseline evaluation. Linear mixed effect models testing the interaction effect of PiB-positivity and time showed that PiB(+) aMCI group had faster decline in MMSE and CDR Sum of Boxes scores than PiB(−) aMCI group after controlling for age, sex and education.

**Table 2:** Longitudinal changes of neuropsychological test scores in PiB(−) and PiB(+) aMCI.

Neuropsychological test	PiB(−) aMCI^*^		PiB(+) aMCI^*^		PiB(−) vs. PiB(+) aMCI^**^	
	β (SE)	*p*	β (SE)	*p*	β (SE)	*p*
Digit span forward	0.14 (0.11)	0.922	0.05 (0.07)	0.570	0.07 (0.12)	0.910
Digit span backward	0.08 (0.08)	0.755	−0.06 (0.08)	0.507	0.16 (0.12)	0.418
Calculation	0.05 (0.15)	0.868	−0.23 (0.08)	0.007	0.33 (0.14)	0.075
K-BNT	0.29 (0.66)	0.806	−1.04 (0.71)	0.205	1.39 (1.10)	0.400
RCFT copy	0.04 (0.35)	0.912	−0.25 (0.30)	0.500	0.27 (0.48)	0.803
SVLT immediate recall	0.30 (0.28)	0.839	−0.66 (0.20)	0.009	0.85 (0.35)	0.089
SVLT delayed recall	−0.24 (0.20)	0.843	−0.34 (0.14)	0.038	0.04 (0.24)	0.932
SVLT recognition	−0.38 (0.21)	>0.999	−0.29 (0.20)	0.196	−0.12 (0.30)	0.848
RCFT immediate recall	−0.44 (0.29)	>0.999	−0.61 (0.30)	0.085	0.25 (0.44)	0.749
RCFT delayed recall	−0.33 (0.36)	0.697	−0.95 (0.30)	0.006	0.62 (0.46)	0.376
RCFT recognition	−0.09 (0.19)	0.828	−0.30 (0.16)	0.111	0.06 (0.26)	0.837
COWAT animal	−0.09 (0.32)	0.825	−0.95 (0.25)	0.002	0.96 (0.39)	0.069
COWAT supermarket	0.23 (0.42)	0.826	−0.70 (0.28)	0.110	1.12 (0.64)	0.244
COWAT phonemic	−0.57 (0.85)	0.780	0.21 (0.83)	0.798	0.02 (1.36)	0.991
Stroop color reading	−2.51 (1.86)	>0.999	−3.78 (1.20)	0.008	1.21 (2.04)	0.855
MMSE	−0.18 (0.07)	0.708	−1.18 (0.18)	<0.001	0.91 (0.28)	0.017
CDR Sum of Boxes	0.10 (0.12)	0.702	0.65 (0.09)	<0.001	−0.48 (0.15)	0.017

PiB(−) aMCI = Pittsburgh compound B negative amnestic mild cognitive impairment; PiB(+) aMCI = Pittsburgh compound B positive amnestic mild cognitive impairment; SE = standard error; K-BNT = Korean version of Boston Naming Test; RCFT = Rey-Osterrieth Figure Test; SVLT = Seoul Verbal Learning Test; COWAT = Controlled Oral Word Association Test; MMSE = Mini-mental Status Examination; CDR = Clinical Dementia Rating. Data are results of linear mixed effect models for neuropsychological scores using age, sex, education, and time interval from baseline evaluation as covariates. ^*^Results of linear mixed models separately performed in PiB(+) or PiB(−) aMCI group using time interval from baseline evaluation as a predictor. ^**^Results of linear mixed models in total aMCI patients using the interaction between group and time interval as a predictor. P values are corrected for multiple comparisons using false discovery rate correction.

### Longitudinal cortical thinning

**Figure 2:**
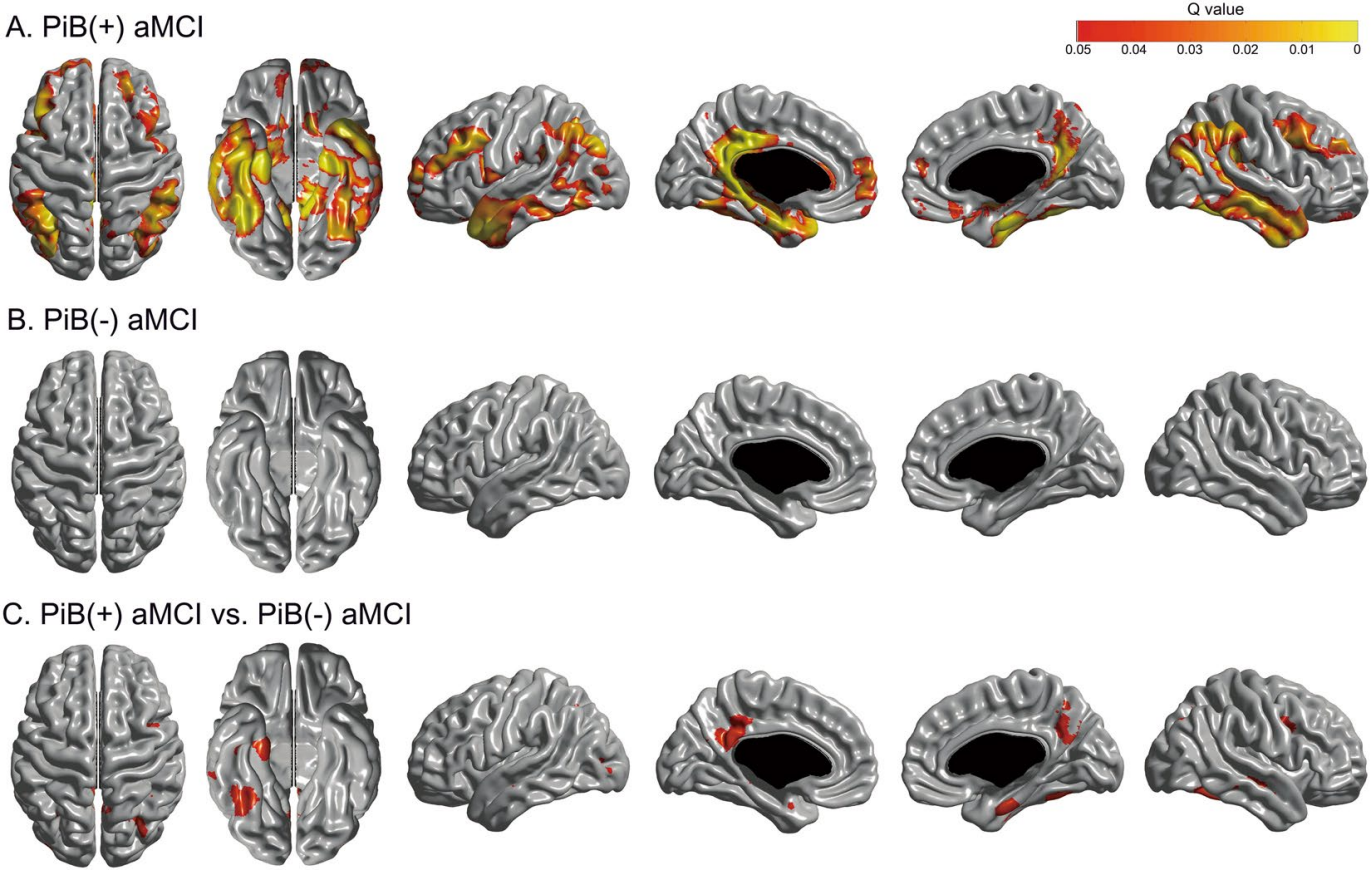
Statistical maps showing cortical regions with longitudinal cortical thinning in (**A**) PiB(+) aMCI group and (**B**) PiB(−) aMCI group, and those with faster rate of cortical thinning in PiB(+) compared PiB(−) aMCI group. PiB(−) aMCI = Pittsburgh compound B negative amnestic mild cognitive impairment; PiB(+) aMCI = Pittsburgh compound B positive amnestic mild cognitive impairment.

Linear mixed effect models separately performed in PiB(+) group and PiB(−) group compared the effect of time interval from baseline MRI on regional W-score with zero and showed that the PiB(+) aMCI group had significant longitudinal cortical thinning in the bilateral medial and lateral temporal, precuneus, posterior cingulate, inferior parietal, and prefrontal regions (Fig. 2). PiB(−) aMCI group did not demonstrate significant longitudinal cortical thinning. There were no regions showing significant increase in cortical thickness over time. Linear mixed effect models testing the interaction effect of PiB-positivity and time on regional W-score showed that PiB(+) aMCI group had a significantly faster rate of cortical thinning than PiB(−) aMCI group in the bilateral medial temporal, right inferolateral temporal, left posterior cingulate, right precuneus, and right prefrontal regions.

### Longitudinal PiB uptake

Twenty-one PiB(+) aMCI and nine PiB(−) aMCI patients had follow-up PiB-PET scans after mean 30.5 ± 6.2 months. Two aMCI patients who had been PiB(−) at baseline converted to PiB(+) at follow up. Mean global and regional PiB uptake at the baseline and follow-up PiB-PET scans are displayed in Supplementary Figure 1. Annual changes of global PiB uptake across all aMCI patients was 0.02 ± 0.05. Annual changes of global PiB uptake in PiB(+) aMCI group and PiB(−) aMCI group were not significantly different [PiB(+) aMCI vs. PiB(−) aMCI = 0.03 ± 0.06 vs. 0.01 ± 0.04, P for independent t-tests = 0.258].

### Subgroups of PiB(−) aMCI

Among 16 PiB(−) aMCI patients who had clinical follow-up, four patients were categorized as depressive aMCI, six as PCC-PiB(+) aMCI, two as aMCI with SVD, and three as aMCI with accelerated aging. One PiB(−) aMCI could not be subcategorized. Demographic and clinical features of subgroups in PiB(−) aMCI patients are summarized in Table 3.

**Table 3:** Comparison of demographic and clinical features of subgroups in PiB(−) aMCI.

	Depressive aMCI	PCC-PiB(+) aMCI	aMCI with SVD	Accelerated aging aMCI	Undetermined
N	4	6	2	3	1
Age: years	70.3 ± 9.5	73.3 ± 3.8	74.0 ± 9.9	69.7 ± 6.4	67
Gender, female	2 (50.0)	4 (66.7)	2 (100.0)	0	1 (100.0)
Education, years	6.5 ± 4.9	12.7 ± 5.8	7.5 ± 2.1	17.0 ± 5.6	16.0
Geriatric Depression Scale	25.0 ± 5.7	10.8 ± 5.3	12.0 ± 2.8	12.3 ± 7.1	15.0
*APOE4* carrier	0/4	0/6	0/2	0/3	0/1
Baseline PiB retention	1.32 ± 0.07	1.41 ± 0.06	1.26 ± 0.01	1.18 ± 0.22	1.31
Annual global PiB retention ratio change^*^	−0.01 ± 0.03	0.02 ± 0.02	−0.06	0.04 ± 0.05	—
Conversion to PiB(+)^*^	0	2 (66.7)	0	0	0
With clinical follow-up	4	6	2	3	1
Follow-up duration, years	4.0 ± 2.7	2.9 ± 1.6	4.3 ± 1.0	4.9 ± 0.1	3.2
Progression to dementia	0	3 (50.0)	1 (50.0)	0	0

*APOE4* = *apolipoprotein E4*; aMCI = amnestic mild cognitive impairment; PiB(−) aMCI = Pittsburgh compound B negative amnestic mild cognitive impairment; PiB(+) aMCI = Pittsburgh compound B positive amnestic mild cognitive impairment; PCC-PiB(+) aMCI = posterior cingulate cortex Pittsburgh Compound B positive amnestic mild cognitive impairment; SVD = small vessel disease. Data are expressed in mean ± standard deviation or number (percentage). ^*^Three of four depressive aMCI, three of six PCC-PiB(+) aMCI, one of two aMCI with SVD, two of three accelerated aging aMCI, and none of one undetermined aMCI patients performed follow-up PiB-PET scans.

Three of the six PCC-PiB(+) aMCI patients progressed to dementia (one AD and two PSPS patients). Demographic, clinical and imaging features of six PCC-PiB(+) aMCI patients are presented in Table 4. Two of three PCC-PiB(+) aMCI patients who underwent follow-up PiB-PET had global PiB uptake >1.5 at the follow-up PiB-PET. Among the two PCC-PiB(+) aMCI patients who exhibited global PiB uptake >1.5 at follow-up, one progressed to AD while the other one remained as aMCI. One of the two aMCI with SVD converted to subcortical vascular dementia.

**Table 4:** Demographic, imaging, and clinical features of six PCC-PiB(+) aMCI patients.

Case	Age	Sex	Associated symptoms	Baseline neuropsychological features^*^	Baseline PiB retention ratio		Follow-up PiB retention ratio		Progression
					Global	PCC	Global	PCC	
1	78	F	Amnesia, word finding difficulty	Memory, Frontal, Memory, Language	1.30	1.54	NA	NA	Drop-out
2	68	F	Amnesia, word finding difficulty	Memory, Frontal	1.47	1.60	1.52	1.68	aMCI
3	72	F	Amnesia, word finding difficulty, anxiety	Memory, Language	1.41	1.62	1.57	1.86	AD
4	73	M	Amnesia, word finding difficulty, dyscalculia, apathy, parkinsonism	Memory, Frontal, Language, Visuospatial	1.43	1.59	NA	NA	PSPS
5	74	F	Amnesia only	Memory, Frontal	1.40	1.53	NA	NA	Drop-out
6	76	M	Amnesia only	Memory, Frontal, Language	1.46	1.58	1.44	1.57	PSPS

AD = Alzheimer’s disease; aMCI = amnestic mild cognitive impairment; DM = diabetes mellitus; F = female; M = male; NA = not applicable; PCC = posterior cingulate cortex; PCC-PiB(+) aMCI = posterior cingulate cortex Pittsburgh Compound B positive amnestic mild cognitive impairment; PSPS = progressive supranuclear palsy syndrome. ^*^Impaired cognitive domains are listed. Cognitive function was considered abnormal when scores in the relevant neuropsychological tests were below 1.0 SD of the norm.

**Figure 3:**
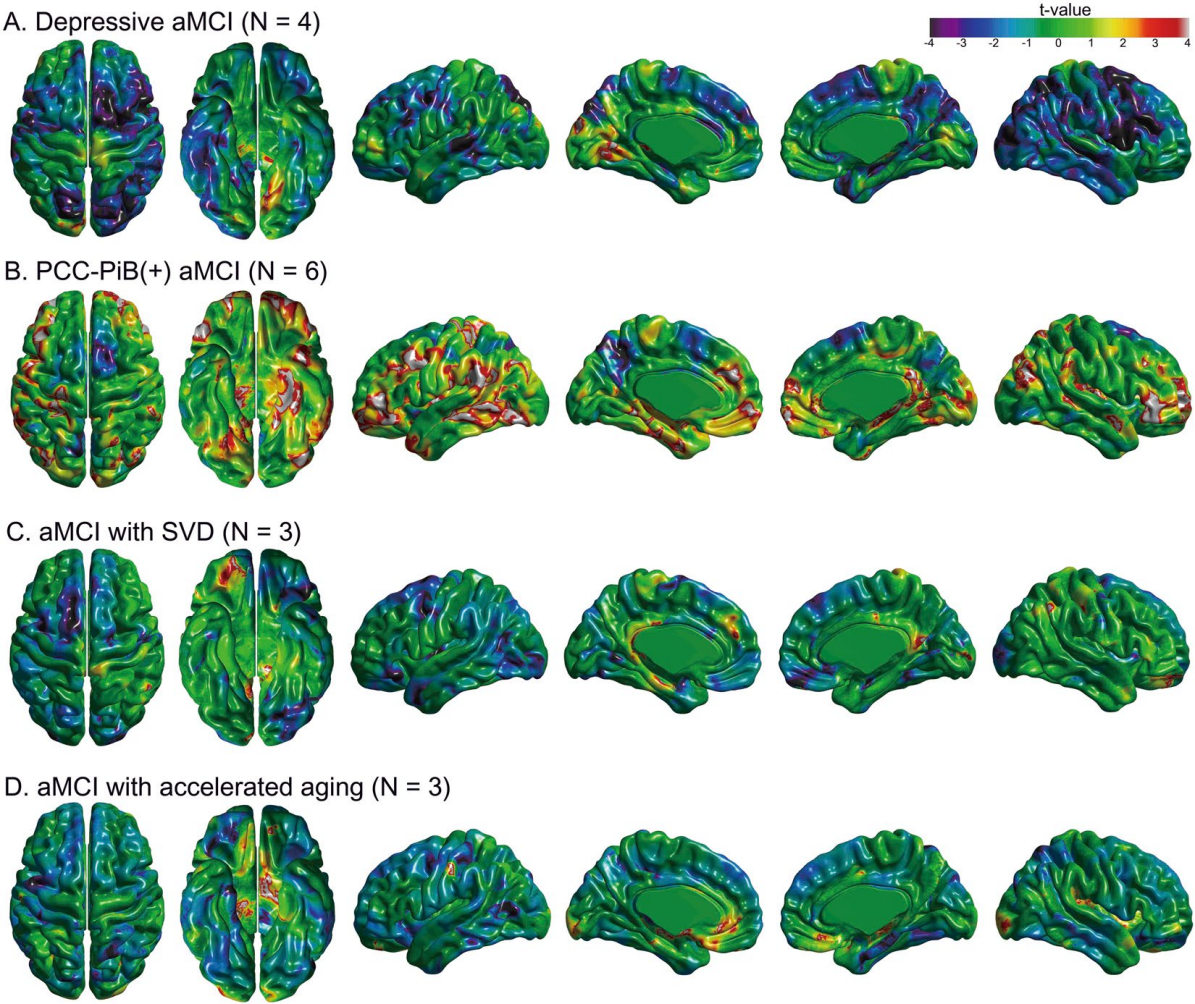
Pattern of longitudinal cortical thinning according to the subgroups of PiB(−) aMCI. Regional maps comparing longitudinal changes of W-scores with zero in (**A**) depressive aMCI with Geriatric Depression Scale >18, (**B**) PCC-PiB(+) aMCI with PiB uptake >1.5 in the PCC, (**C**) aMCI with SVD, and (**D**) aMCI with accelerated aging. PiB(−) aMCI = Pittsburgh compound B negative amnestic mild cognitive impairment; PiB(+) aMCI = Pittsburgh compound B positive amnestic mild cognitive impairment; PCC = posterior cingulate cortex; SVD = small vessel disease.

To evaluate the topographical tendency (t value >2) of longitudinal cortical thinning in PiB(−) aMCI subgroups, we performed linear mixed effect models comparing the effect of time on regional W-score with zero in respective PiB(−) aMCI subgroups (Fig. 3). The degree of longitudinal cortical thinning was most prominent in PCC-PiB(+) aMCI and localized to the bilateral medial temporal, left PCC, left superior temporal-inferior parietal, bilateral prefrontal and anterior cingulate regions. On the other hand, in PCC-PiB(+) aMCI group, a trend of longitudinal cortical thickening was noted in the bilateral precuneus regions. In aMCI with SVD, a trend of longitudinal cortical thinning was noted in the right basal frontal, middle and posterior cingulate, and left medial temporal regions. In aMCI with accelerated aging, trend of longitudinal cortical thinning was observed in the bilateral basal frontal, right posterior insula, and left occipital regions.

## Discussion

In this prospective cohort study, we report a longitudinal course of aMCI patients with or without significant cerebral PiB uptake in terms of clinical, neuropsychological, and brain morphological changes. As a group, PiB(+) aMCI patients showed a risk of progression to dementia about three times greater than that of PiB(−) aMCI patients. PiB(+) aMCI patients also showed significant longitudinal cortical thinning in several AD-prone cortical regions. We did not observe any regions demonstrating significant cortical thinning over time in the PiB(−) aMCI group. Among PiB(−) aMCI patients, more subjects in the PCC-PiB(+) aMCI subgroup converted to globally amyloid positive and dementia than in other groups, though our numbers prevented us from testing this association statistically. Taken together, our findings suggested that amyloid PET is important in prediction of prognosis in aMCI patients. Furthermore, we need to pay more attention to the subgroup of PiB(−) aMCI with increased regional PiB uptake especially in the PCC.

We found that PiB(+) aMCI converted to dementia a 3.2 times the rate of PiB(−) aMCI. Our finding is consistent with previous studies^14,15^, which showed that amyloid-positive aMCI patients had a HR of 3.2 (95% CI, 1.4–7.1), and 50% of amyloid-positive aMCI patients progressed to dementia in 2 years^14^. Our conclusion that amyloid positivity in aMCI is one of the most important predictors of AD dementia is supported by another observation: specifically, we found that PiB(+) aMCI showed longitudinal decline in cognitive scores mainly involving memory and semantic fluency tests, which are neuropsychological hallmarks of AD^16,17^. We also found that PiB(+) aMCI patients showed a longitudinal cortical thinning pattern reminiscent of AD^18^ involving the medial temporal cortices, lateral temporal and parietal cortices, precuneus, and PCC.

In the present study, four of 16 PiB(−) aMCI patients progressed to dementia (progression rate = 6.3%/year). These PiB(−) aMCI patients progressed to various causes of dementia including PSPS, vascular dementia, and AD. Considering that the PiB(−) aMCI group showed significant cortical and hippocampal atrophy compared to control subjects in our previous study, PiB(−) aMCI could be regarded to have neurodegeneration just like suspected non-Alzheimer disease pathophysiology (SNAP)^19,20^. Previous studies have reported that around 17~35% of MCI patients have SNAP, and that these individuals progress to AD as well as non-AD dementia with annual rates ranging from 12.4~25.5%/year^21–25^. Previous studies have also shown that aMCI without significant amyloidosis could progress to frontotemporal dementia, Lewy body dementia, vascular dementia, Parkinson’s disease dementia, PSPS, and even AD^15,25,26^. Consistent with previous studies of aMCI with SNAP, our findings therefore suggested that PiB(−) aMCI group might be composed of heterogeneous entities. We also found that there was no *APOE4* carrier among PiB(−) aMCI patients. This finding is consistent with a previous study based on ADNI dataset which reported the relative paucity of APOE4 carriage in PiB(−) aMCI group compared to PiB(+) aMCI group^27^.

Another noteworthy finding was that PCC-PiB(+) aMCI subgroup, which were globally amyloid negative but showed increased uptake just in the PCC, are likely to convert to globally amyloid positive, suggesting that these patients have subthreshold cerebral amyloid deposition. Because the cut-offs to determine global PiB-positivity is somewhat arbitrary^28,29^, we cannot exclude the possibility that these PCC-PiB(+) aMCI patients have significant amyloid deposition in their brain. The clinical significance of increased uptake specifically in the PCC is very important, but rarely studied. The PCC is one of the first regions to accumulate amyloid in Alzheimer’s disease and is thought of as an epicenter of amyloid burden^7,30^. Previous studies have also shown that converters to AD had higher PiB retention specifically in the PCC compared to non-converters^31–33^. Similar to amyloid PET, FDG PET shows abnormal metabolism in the PCC even in the prodromal AD^34,35^. Therefore, our findings suggested that regional Aβ accumulation could have substantial effects on the clinical course of our PCC-PiB(+) aMCI patients. In fact, two of three PCC-PiB(+) aMCI patients converted to globally PiB(+) at PET follow up.

Interestingly, PCC-PiB(+) aMCI group showed a trend of longitudinal cortical thickening in the bilateral precuneus regions. A previous study reported that temporal lobe volume is higher in healthy control subjects with significant β-amyloid deposition compared to those without^36^; and other studies showed a hippocampal hypertrophy of the neuronal nuclei and cell bodies in the brains of normal elderly with β-amyloid plaques compared to those without β-amyloid plaques at autopsy^37,38^. Although the precuneus regions have not been investigated in these studies, our finding of longitudinal cortical thickening in the precuneus may reflect a compensatory response to cerebral β-amyloid deposition.

Considering that the threshold for global PiB-positivity is somewhat arbitrary^28,29^, we defined PiB-positivity differently according to cut-offs of global PiB uptake ratio (from 1.35 to 1.60) and further performed Cox regression analyses using PiB-positivity and baseline age as predictors (Supplementary Table 1). According to these analyses, the cut-off of global PiB uptake ratio for best accuracy in Cox regression was 1.41. These results are consistent with a previous study showing that lowering the threshold value of PiB-PET uptake resulted in higher sensitivity while not compromising specificity^39^.

The strengths of our study are its prospective setting, standardized imaging protocols, and detailed clinical evaluation during the follow-up. However, there are several limitations in our study. First, we could not perform pathology confirmation through autopsy. Some clinical diagnosis could be misclassified. Second, because of the small sample size in the subgroup of PiB(−) aMCI, we could not provide the statistical comparison of longitudinal changes in cognition and cortical thinning. Third, because aMCI patients were recruited from a memory clinic, our results may not be generalizable to other settings. Finally, as the recruitment of study subjects was made before 2011, we could not apply the revised National Institute on Aging-Alzheimer’s Association (NIA-AA) criteria published in 2011^40^. However, when we reviewed medical records, all the aMCI patients met the core clinical criteria of the revised diagnostic NIA-AA criteria for MCI due to AD^40^. Nonetheless, our findings suggested that amyloid PET is important in prediction of prognosis in aMCI patients. Furthermore, we need to pay more attention to the subgroup of PiB(−) aMCI with increased regional PiB uptake, especially in the PCC.

## Methods

### Patients

Between February 2009 and July 2011, we recruited 47 aMCI patients who underwent PiB PET and were clinically followed up from the Samsung Medical Center memory clinics (Seoul, Republic of Korea). All aMCI patients met the following diagnostic criteria based on the guidelines proposed by Petersen *et al*.^41^: (1) subjective memory complaint by subjects or caregivers; (2) normal general cognitive function defined by scores on the Mini-Mental State Examination (MMSE) greater than −1.0 SD of the norms for age- and education-matched normal subjects; (3) normal activities of daily living (ADL), as judged clinically as well as by the ADL scale described later; (4) objective memory decline below −1.0 SD on either verbal or visual memory tests; (5) and not demented. These diagnostic criteria and cut-off value for memory impairment have been used in previous studies^9,42,43^.

All the patients underwent clinical interviews, a neurological examination, neuropsychological tests, brain MRI, and PiB PET imaging at baseline. ADL was assessed by the Seoul Instrumental ADL, which was described in a previous study^42^. Depressive symptoms were evaluated with the Geriatric Depression Scale^44^. Participants with current or past neurologic or psychiatric illnesses including schizophrenia, major depressive disorders, epilepsy, brain tumors, encephalitis, and severe head trauma were excluded. Patients with severe hearing or visual loss, aphasia, severe cardiac disorders, malignancies, and hepatic or renal disorders were further excluded. Blood tests included a complete blood count, blood chemistry tests, vitamine B12/folate, syphilis serology, and thyroid function tests. On brain magnetic resonance imaging (MRI), patients with structural lesions, such as tumors, traumatic brain injuries, or hydrocephalus were excluded. We also excluded the patients who had prominent atypical presentations of language, behavioral changes, or parkinsonism at baseline examination and patients with severe periventricular or deep white matter ischemia (as indicated by a score more than 3 on the Fazekas ischemia scale)^45^.

In our previous study, 48 aMCI patients were categorized into 32 (66.7%) PiB(+) aMCI and 16 (33.3%) PiB(−) aMCI patients. PiB(−) aMCI patients were further subcategorized into the following subgroups: depressive aMCI, PCC-PiB(+) aMCI, aMCI with small vessel disease (SVD) and aMCI with accelerated aging groups, which were described in a previous study in detail^9^. Depressive aMCI had Geriatric Depression Scale >18; PCC-PiB(+) aMCI with PiB uptake ratio >1.5 in the PCC; aMCI with SVD had a moderate degree of WMH (periventricular WMH ≥10 mm and 10 mm ≤ deep WMH <25 mm); and aMCI with accelerated aging had vascular risk factors including hypertension or diabetes mellitus without any of the above features. One PiB(+) aMCI patient who did not have clinical follow-up was excluded in the current study.

We obtained written, informed consent from each participant. This study protocol was approved by the Institutional Review Board of Samsung Medical Center. All methods were performed in accordance with relevant guidelines and regulations. This manuscript does not contain information or image that can lead to identification of a study participant.

### Neuropsychological assessments

All participants underwent a standardized neuropsychological battery called the Seoul Neuropsychological Screening Battery (SNSB), which includes tests for attention, language, praxis, four elements of Gerstmann syndrome, visuoconstructive function, verbal and visual memory, and frontal/executive function^46^. The scorable tests included digit span forward and backward, the Korean version of the Boston Naming Test (K-BNT), the Rey-Osterrieth Complex Figure Test (RCFT: copying, immediate recall, 20-minute delayed recall, and recognition), Seoul Verbal Learning Test (SVLT: immediate recall, 20-minute delayed recall, and recognition), phonemic and semantic Controlled Oral Word Association Test (COWAT), and the Stroop test (word and color reading). These tests were considered abnormal when the scores were below −1.0 SD of the norms for the age- and education-matched normal subjects. Memory function was considered abnormal when the score for the delayed recall items of the SVLT or RCFT was below the −1.0 SD of the norm.

### PiB-PET acquisition

All aMCI patients underwent [^11^C] PiB-PET at Samsung Medical Center or Asan Medical Center within three months before or after the neuropsychological assessments. Discovery STe PET/computed tomography (CT) scanners (GE Medical Systems, Milwaukee, WI) were used in a 3-dimensional (3D) scanning mode examining 35 slices with 4.25 mm thickness that spanned the entire brain. The [^11^C] PiB was injected into an antecubital vein as a bolus with a mean dose of 420 MBq. Sixty minutes after the injection, a CT scan was performed for attenuation correction. Then, a 30-minute emission static PET scan was initiated.

### PET data analysis

Data processing was performed using SPM version 5 under Matlab 6.5 (Mathworks, Natrick, MA). PiB-PET images were co-registered to individual MRIs and normalized to a T1-weighted MRI template. The quantitative regional values of PiB uptake on the spatially normalized PiB images were acquired using volumes of interest (VOIs) drawn from the automated anatomical labeling (AAL) atlas^47^. To measure PiB uptake, the cerebral cortical region to cerebellum uptake ratio was used. The global PiB uptake ratio was calculated from the volume-weighted average uptake ratio of 28 bilateral cortical VOIs. VOIs were selected using the AAL atlas, and include the bilateral frontal (superior and middle frontal gyri; medial portion of the superior frontal gyrus; opercular and triangular portions of the inferior frontal gyrus; supplementary motor area; orbital portion of the superior, middle, and inferior orbital frontal gyri; rectus and olfactory cortex), PCC, parietal (superior and inferior parietal parietal gyri, supramarginal and angular gyri, and precuneus), lateral temporal (superior, middle, and inferior temporal gyri, an Heschl gyri), and occipital (superior, middle, and inferior occipital gyri, cuneus, calcarine fissure, and lingual gyrus, and fusiform gyrus). Patients were considered PiB-positive if their global PiB uptake ratio was two standard deviations or higher than the mean of global PiB uptake ratio in normal controls, which was 1.5^48^.

### MRI acquisition

All aMCI patients underwent baseline MRI with a 3.0 Tesla MRI scanner (Achieva; Philips Medical Systems, Best, The Netherlands) at Samsung Medical Center within three months before or after the baseline neuropsychological assessments. 3D T1 turbo field echo MR images were acquired with following parameters: sagittal slice thickness, 1.0 mm, over contiguous slices with 50% overlap; no gap; repetition time (TR) of 9.9 ms; echo time (TE) of 4.6 ms; flip angle of 8°; and matrix size of 240 × 240 pixels, reconstructed to 480 × 480 over a field of view 240 mm.

### Cortical thickness data analysis

T1-weighted images were processed using the standard Montreal Neurological Institute anatomic pipeline. Further methods for imaging preprocessing are detailed in eMethods. Based on the cortical thickness data of 54 subjects with normal cognition, W-scores of cortical thicknesses were calculated with more positive W-score representing more severe cortical thinning considering patients’ age, sex and ICV.

### Construction for W-scores map

We carried out the W-scores map to identify the degree of a patient’s cortical thinning using the cortical thickness data of 54 cognitively normal healthy subjects as a reference. These 54 control subjects underwent brain MRI using the same MRI scanner with aMCI patients and information about age, gender, education and ICV was provided in Supplementary Table 2. There were no differences between 47 aMCI patients and 54 cognitively normal subjects in terms of age, gender and years of education. The detailed concept and computation of W-scores are available elsewhere^49^. In this study, W-scores maps were computed vertex-wise on the surface model and voxel-wise on the skeletonized volume of each imaging data as follows: W-score = [(patient’s raw value) – (value expected in the control group for the patient’s age, sex, and intracranial volume (ICV))]/SD of the residuals in controls. ICV was defined as the sum of gray matter, white matter, and cerebrospinal fluid (CSF) volume. W-scores are similar to Z-scores, with a mean value of 0 and SD of 1 in the control group, and values of +1.65 and −1.65 correspond to the 95^th^ and 5^th^ percentiles, respectively. However, unlike Z-scores, they are adjusted for specific covariates such as age, sex, and ICV. To avoid confusion, we inverted the W-scores such that positive values indicate thinner cortex.

### Clinical follow-up

A total of 47 patients were annually evaluated for three years through clinical interviews, neuropsychological tests and brain MRI. Clinical follow-up for progression to dementia was performed until April 2015. All patients had clinical follow-up [follow-up duration, mean (SD), 3.6 (1.3) years]. The diagnosis of dementia was based on the criteria from the Diagnostic and Statistical Manual of Mental Disorders (fourth edition) and required evidence of impairment in social or occupational functions confirmed by the Seoul Instrumental ADL. For the diagnosis of probable AD, PSPS and subcortical vascular dementia, we applied standard research criteria for dementia syndromes^50–52^.

### Follow-up evaluations with neuropsychological tests, MRI and PiB-PET

**Figure 4:**
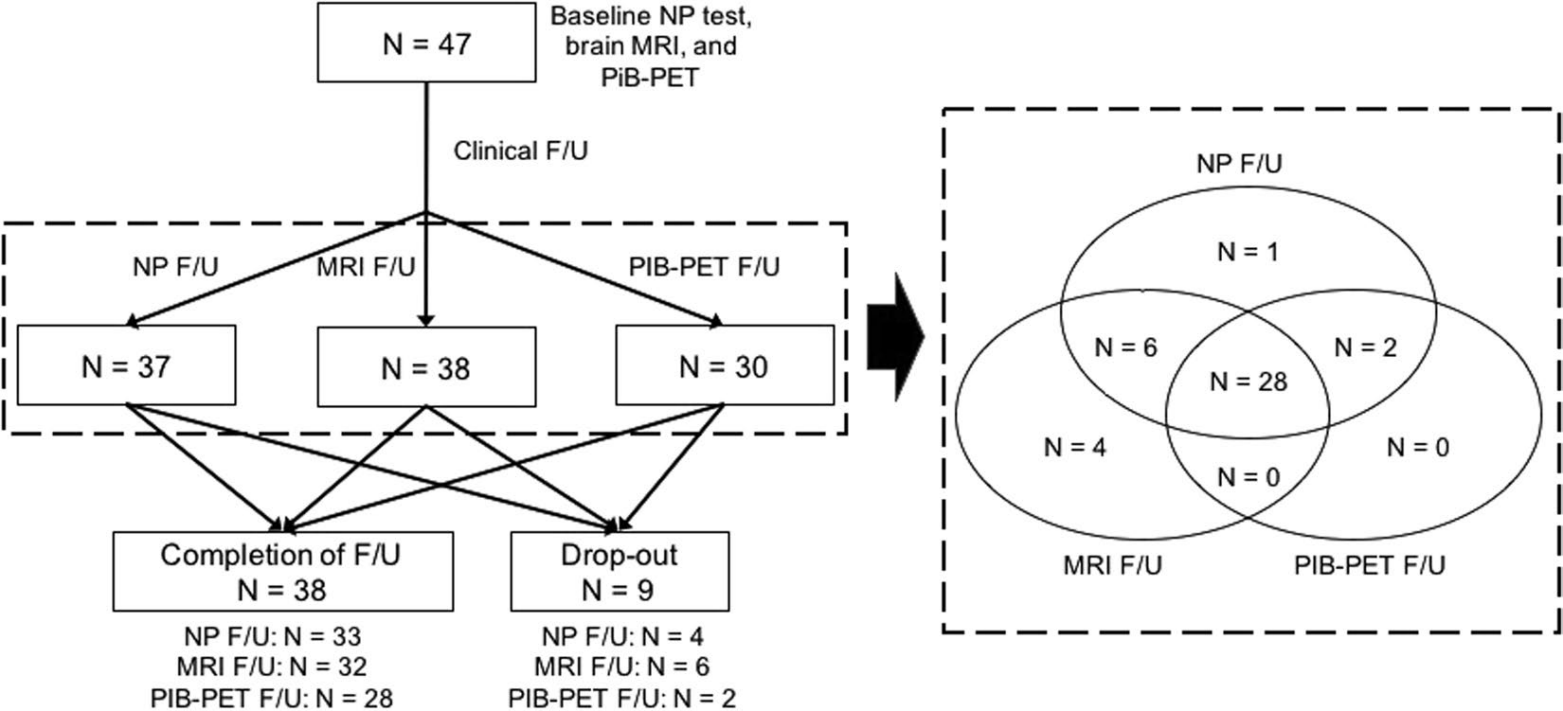
Flow charts showing the number of subjects that completed each portion of the protocol including clinical, neuropsychological and imaging follow-up. F/U = follow-up; NP = neuropsychological; MRI = magnetic resonance imaging; PiB-PET = Pittsburgh compound B positron emission tomography.

A total of 37 patients had follow-up neuropsychological tests (17 patients had one follow-up neuropsychological test, 12 patients had two follow-up tests, four patients had three follow-up tests, and four patients had five follow-up tests). Thirty-eight aMCI patients had follow-up brain MRI, and 30 patients had follow-up PiB-PET scans. Figure 4 shows the number of subjects that completed each portion of the protocol including clinical, neuropsychological and imaging follow-up. We also compared 38 patients who completed the follow-up and nine patients with drop-out (Supplementary Table 3). There were no differences in terms of baseline age, gender, education, and follow-up duration, APOE4 carriage, and vascular risk factors. Patients who completed the follow-up had higher baseline global PiB retention ratio and were more likely to have baseline PiB-positivity and follow-ups for neuropsychological tests and PiB-PET than those with drop-out.

### Statistical analyses

All statistical analyses were performed in patients who had clinical follow-up. Demographic features of PiB(+) aMCI and PiB(−) aMCI groups were compared with independent t-tests and chi-square tests as appropriate. Cox regression models were used to compare the risks of progression to dementia between PiB(+) aMCI and PiB(−) aMCI groups after controlling for age, sex, and education. Time to the event was defined as the time from PiB-PET scanning time to the follow-up visit at which a first-time diagnosis of dementia was made. Patients who did not progress to dementia were treated as censored observations from the time of their final follow-up visit.

Longitudinal changes in cognitive scores in two aMCI groups were compared with linear mixed effect models using age, sex, education, and time interval from baseline neuropsychological tests as covariates, and the interaction of PiB(+) and the time interval as a predictor. To find the trend of cognitive changes in each group, linear mixed models were separately performed in each group using age, sex, and education as covariates, and time interval from baseline evaluation as a predictor. Because multiple cognitive scores were used for comparison, correction for multiple comparisons was performed by false discovery rate correction.

Rate of regional longitudinal cortical thinning of PiB(+) aMCI group and PiB(−) aMCI groups were compared using linear mixed effect models on a vertex-by-vertex basis using time interval from baseline MRI and PiB(+) as covariates and the interaction of PiB(+) and the time interval from baseline MRI as a predictor. In each PiB group, the rate of longitudinal cortical thinning was compared to zero with linear mixed effect models using the time interval from baseline MRI as a predictor. All cortical thickness analyses used W-score and no further adjustments were performed. Because the cortical surface model contained 81,924 vertices, correction for multiple comparisons was performed by false discovery rate correction at a corrected probability value of 0.05.

Demographic features, neuropsychological test scores, and risks for progression to dementia were analyzed using the Statistical Package for the Social Sciences 18.0 (SPSS Inc, Chicago, IL, USA), and cortical thickness data were analyzed using Matlab 7.11 for Windows (MathWorks, Natrick, MA, USA).

## Electronic supplementary material

Supplementary materials
