# Supplementary material for: Longitudinal outcomes of amyloid positive versus negative amnestic mild cognitive impairments: a three-year longitudinal study
Source: Sci Rep. 2018 Apr 3;8:5557. doi: 10.1038/s41598-018-23676-w (PMC5883059; doi:10.1038/s41598-018-23676-w)
Supplement: Supplementary file 1 — Supplementary materials [file 41598_2018_23676_MOESM1_ESM.doc]

**Longitudinal outcomes of amyloid positive versus negative amnestic mild cognitive impairments: a three-year longitudinal study**

Byoung Seok Ye, MD,^1^ Hee Jin Kim, MD,^2^ Yeo Jin Kim, MD,^3^ Na-Yeon Jung, MD,^4^ Jin San Lee, MD,^5^ Juyoun Lee, MD,^2^ Young Kyoung Jang, MD,^2^ Jin-ju Yang, MD,^6^ Jong-Min Lee, PhD,^6^ Jacob W. Vogel,^7^ Duk L. Na, MD,^2^ Sang Won Seo, MD^2^

^1^ Department of Neurology, Yonsei University College of Medicine, Seoul 03722, Korea

^2^ Department of Neurology, Samsung Medical Center, Sungkyunkwan University School of Medicine, Seoul 06351, Korea

^3^ Department of Neurology, Chuncheon Sacred Heart Hospital, Hallym University College of Medicine, Chuncheon, Korea

^4^ Department of Neurology, Pusan National University Hospital, Pusan National University School of Medicine and Medical Research Institute, Busan, South Korea

^5^ Department of Neurology, Kyung Hee University Hospital, Seoul, Korea

^6^ Department of Biomedical Engineering, Hanyang University, Seoul, South Korea

^7^ Montreal Neurological Institute, McGill University, Montrèal, Quebec, Canada

Supplementary table 1. ROC curve analysis using differently defined PiB-positivity.

| Cut-off | Hazard ratio (95% CI) | Accuracy (95% CI) |
| --- | --- | --- |
| 1.35 | 7.22 (0.98-53.37) | 0.52 (0.33-0.71) |
| 1.36 | 7.22 (0.98-53.36) | 0.52 (0.33-0.71) |
| 1.37 | 9.10 (1.23-67.28) | 0.56 (0.37-0.74) |
| 1.38 | 9.10 (1.23-67.28) | 0.56 (0.37-0.74) |
| 1.39 | 9.10 (1.23-67.28) | 0.56 (0.37-0.74) |
| 1.40 | 11.28 (1.52-83.51) | 0.61 (0.43-0.79) |
| 1.41 | 11.28 (1.52-83.51) | 0.61 (0.43-0.79) |
| 1.42 | 5.97 (1.41-25.36) | 0.55 (0.37-0.73) |
| 1.43 | 5.97 (1.41-25.36) | 0.55 (0.37-0.73) |
| 1.44 | 3.82 (1.14-12.77) | 0.51 (0.33-0.69) |
| 1.45 | 3.82 (1.14-12.77) | 0.51 (0.33-0.69) |
| 1.46 | 3.00 (1.03-8.73) | 0.48 (0.30-0.65) |
| 1.47 | 3.00 (1.03-8.73) | 0.48 (0.30-0.65) |
| 1.48 | 3.61 (1.24-10.51) | 0.51 (0.33-0.69) |
| 1.49 | 3.61 (1.24-10.51) | 0.51 (0.33-0.69) |
| 1.50 | 3.61 (1.24-10.51) | 0.51 (0.33-0.69) |
| 1.51 | 3.61 (1.24-10.51) | 0.51 (0.33-0.69) |
| 1.52 | 4.39 (1.50-12.85) | 0.55 (0.38-0.72) |
| 1.53 | 4.39 (1.50-12.85) | 0.55 (0.38-0.72) |
| 1.54 | 4.39 (1.50-12.85) | 0.55 (0.38-0.72) |
| 1.55 | 4.39 (1.50-12.85) | 0.55 (0.38-0.72) |
| 1.56 | 4.39 (1.50-12.85) | 0.55 (0.38-0.72) |
| 1.57 | 3.46 (1.30-9.23) | 0.52 (0.34-0.69) |
| 1.58 | 3.46 (1.30-9.23) | 0.52 (0.34-0.69) |
| 1.59 | 3.46 (1.30-9.23) | 0.52 (0.34-0.69) |
| 1.60 | 3.46 (1.30-9.23) | 0.52 (0.34-0.69) |

PiB = Pittsburgh compound B; ROC = receiver operating characteristic; CI = confidence interval. Results of Cox regression analyses for dementia conversion using PiB-positivity that was defined various global PiB uptake ratios (from 1.35 to 1.60) and baseline age as predictors.

Supplementary table 2. Information about 54 cognitively normal subjects for the construction of W-score.

|  | Features |
| --- | --- |
| N | 54 |
| Age | 68.2 ± 5.1 |
| Gender, female | 40 (74.1) |
| Education, years | 11.5 ± 4.7 |
| Intracranial volume | 1349.2 ± 110.4 |

Data are expressed in mean ± standard deviation or number (percentage).

Supplementary table 3. Comparison of demographic and clinical features of patients with drop-out and those without.

|  | Without drop-out | With drop-out | P value |
| --- | --- | --- | --- |
| Number | 38 | 9 |  |
| Baseline age | 70.1 ± 8.2 | 73.1 ± 6.5 | 0.301 |
| Gender |  |  |  |
| Education | 12.7 ± 4.5 | 13.8 ± 4.6 | 0.509 |
| Baseline PiB uptake | 1.96 ± 0.48 | 1.51 ± 0.42 | 0.013 |
| Baseline PiB-positivity | 28 (73.7) | 3 (33.3) | 0.045 |
| Follow-up duration, years | 3.8 ± 1.4 | 2.8 ± 1.5 | 0.079 |
| *APOE4* | 16 (44.4) | 3 (33.3) | 0.712 |
| Vascular risk factors |  |  |  |
| DM | 4 (10.5) | 1 (11.1) | > 0.999 |
| HTN | 15 (39.5) | 5 (55.6) | 0.465 |
| Hyperlipidemia | 6 (15.8) | 4 (44.4) | 0.081 |
| Heart disease | 5 (13.2) | 2 (22.2) | 0.605 |
| Stroke history | 2 (5.3) | 0 | > 0.999 |
| NP follow-up | 33 (86.8) | 4 (44.4) | 0.013 |
| MRI follow-up | 32 (84.2) | 6 (66.7) | 0.344 |
| PiB-PET follow-up | 28 (73.7) | 2 (22.2) | 0.007 |

PiB = Pittsburgh compound B; DM = diabetes mellitus; HTN = hypertension; NP = neuropsychological; MRI = magnetic resonance imaging; PiB-PET = Pittsburgh compound B positron emission tomography. Results of independent t-tests or chi-square tests as appropriate. Data are expressed in mean ± standard deviation or number (percentage).

Supplementary figure 1. Mean global (A) and regional (B-E) PiB retention ratios at the baseline and follow-up PiB-PET scans. Blue bars represent baseline PiB retention ratios and green bars represent follow-up PiB retention ratios.


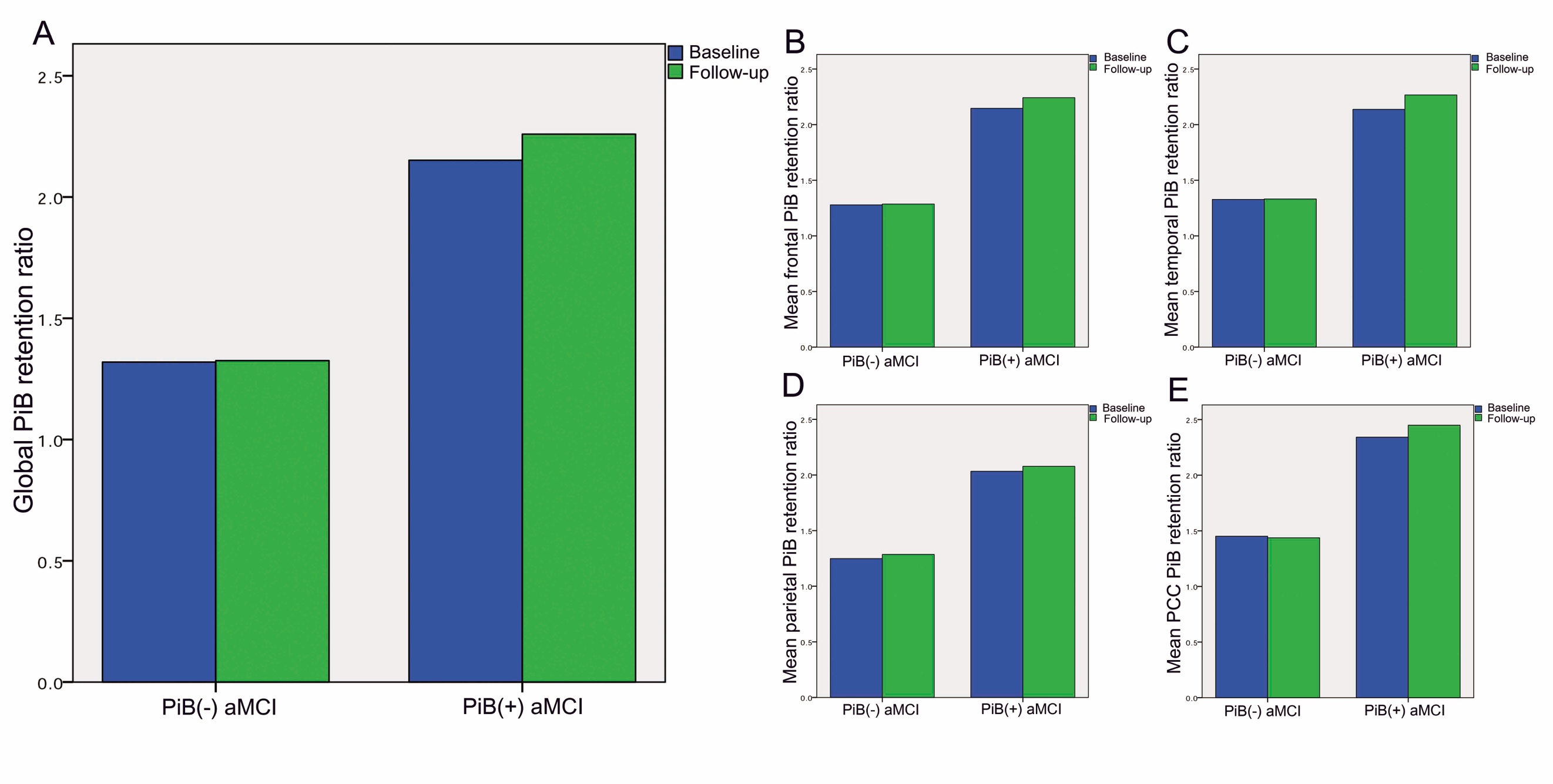


PiB(-) aMCI = Pittsburgh compound B negative amnestic mild cognitive impairment; PiB(+) aMCI = Pittsburgh compound B positive amnestic mild cognitive impairment

**eMethod**

**Image processing for cortical thickness measurement**

With a linear transformation, native MRI images were registered into a standardized stereotaxic space^1^. The N3 algorithm was used to correct the images for intensity-based non-uniformities^2^ caused by the nonhomogeneities in the magnetic field. Then, the registered and corrected images were classified into white matter, gray matter, CSF, and background, using a 3D stereotaxic brain mask and the Intensity-Normalized Stereotaxic Environment for Classification of Tissues (INSECT) algorithm^3^. The surfaces of the inner and outer cortex were automatically extracted using the Constrained Laplacian-Based Automated Segmentation with Proximities (CLASP) algorithm^4^.

Cortical thickness values were calculated in the native space rather than Talairach space because of the limitations in linear stereotaxic normalization. As we transformed MR volumes in native space into stereotaxic space with a linear transformation matrix, the inverse transformation matrix was applied to the cortical thickness models to reconstruct them in native space^5^. Cortical thickness was defined as the Euclidean distance between the linked vertices of the inner and outer surfaces^4^. The thickness value was spatially normalized using surface-based two-dimensional registration with a sphere-to-sphere warping algorithm. Thus, the vertices of each subject were nonlinearly registered to a standard surface template^6,7^. Cortical thickness was subsequently smoothed using a surface-based diffusion kernel in order to increase the signal-to-noise ratio. We chose 20 mm full-width at half-maximum as the kernel size to maximize statistical power while minimizing false positives^8^.

**eReferences**

1 Collins, D. L., Neelin, P., Peters, T. M. & Evans, A. C. Automatic 3D intersubject registration of MR volumetric data in standardized Talairach space. *J Comput Assist Tomogr* **18**, 192-205 (1994).

2 Sled, J. G., Zijdenbos, A. P. & Evans, A. C. A nonparametric method for automatic correction of intensity nonuniformity in MRI data. *IEEE Trans Med Imaging* **17**, 87-97, doi:10.1109/42.668698 (1998).

3 Zijdenbos, A. *et al.* in *Visualization in Biomedical Computing: 4th International Conference, VBC'96 Hamburg, Germamy, September 22–25, 1996 Proceedings* (eds Karl Heinz Höhne & Ron Kikinis) 439-448 (Springer Berlin Heidelberg, 1996).

4 Kim, J. S. *et al.* Automated 3-D extraction and evaluation of the inner and outer cortical surfaces using a Laplacian map and partial volume effect classification. *Neuroimage* **27**, 210-221, doi:10.1016/j.neuroimage.2005.03.036 (2005).

5 Im, K. *et al.* Gender difference analysis of cortical thickness in healthy young adults with surface-based methods. *Neuroimage* **31**, 31-38, doi:10.1016/j.neuroimage.2005.11.042 (2006).

6 Lyttelton, O., Boucher, M., Robbins, S. & Evans, A. An unbiased iterative group registration template for cortical surface analysis. *Neuroimage* **34**, 1535-1544, doi:10.1016/j.neuroimage.2006.10.041 (2007).

7 Robbins, S., Evans, A. C., Collins, D. L. & Whitesides, S. Tuning and comparing spatial normalization methods. *Med Image Anal* **8**, 311-323, doi:10.1016/j.media.2004.06.009 (2004).

8 Chung, M. K. *et al.* Deformation-based surface morphometry applied to gray matter deformation. *Neuroimage* **18**, 198-213 (2003).
